# Empirical Meropenem Versus Piperacillin/Tazobactam for Critically Ill Adults With Sepsis: Feasibility of a Randomised Trial

**DOI:** 10.1111/aas.70324

**Published:** 2026-08-31

**Authors:** Nick Frørup Meier, Morten Hylander Møller, Maj‐Brit Nørregaard Kjær, Fredrik Sjövall, Marie Warrer Munch, Benjamin Skov Kaas‐Hansen, Hans‐Christian Thorsen‐Meyer, Bodil Steen Rasmussen, Marie Helleberg, Frederik Boëtius Hertz, Theis Lange, Aksel Karl Georg Jensen, Lars Wiuff Andersen, Jakob Steen Andersen, Jens Michelsen, Lars Peter Kloster Andersen, Morten Heiberg Bestle, Anne Craveiro Brøchner, Theis Skovsgaard Itenov, Steffen Christensen, Ronni Thermann Reitz Plovsing, Klaus Ulrik Koch, Anders Møller, Olav Lilleholt Schjørring, Naia Bech Cranø, Kasper Vamdrup‐Andersen, Davide Placido, Tine Sylvest Meyhoff, Carl Thomas Anthon, Aske Tøgern, Milda Grigonyte‐Daraskeviciene, Zemira Engbakken, Anne Witte Kamstrup, Kis Rønn Uhre, Jehad Ahmad Barakji, Rikke Faebo Larsen, Anders Perner, Anders Granholm

**Affiliations:** ^1^ Department of Intensive Care Copenhagen University Hospital—Rigshospitalet Copenhagen Denmark; ^2^ Collaboration for Research in Intensive Care (CRIC) Copenhagen Denmark; ^3^ Department of Clinical Medicine University of Copenhagen Copenhagen Denmark; ^4^ Department of Intensive and Perioperative Care Skåne University Hospital Malmö Sweden; ^5^ Department of Clinical Sciences Lund University Malmö Sweden; ^6^ Department of Anaesthesia and Intensive Care Aalborg University Hospital Aalborg Denmark; ^7^ Department of Clinical Medicine Aalborg University Aalborg Denmark; ^8^ Department of Infectious Diseases Copenhagen University Hospital—Rigshospitalet Copenhagen Denmark; ^9^ Department of Clinical Microbiology Copenhagen University Hospital—Rigshospitalet Copenhagen Denmark; ^10^ Department of Immunology and Microbiology University of Copenhagen Copenhagen Denmark; ^11^ Section of Biostatistics, Department of Public Health University of Copenhagen Copenhagen Denmark; ^12^ Department of Anesthesiology and Intensive Care Medicine Aarhus University Hospital Aarhus Denmark; ^13^ Department of Clinical Medicine Aarhus University Aarhus Denmark; ^14^ Prehospital Emergency Medical Services Aarhus Central Denmark Region Denmark; ^15^ Department of Anaesthesiology and Intensive Care Medicine Odense University Hospital Odense Denmark; ^16^ Department of Anaesthesiology Zealand University Hospital Køge Denmark; ^17^ Department of Intensive Care Copenhagen University Hospital—North Zealand Hillerød Denmark; ^18^ Department of Anaesthesiology and Intensive Care Lillebælt Hospital Kolding Denmark; ^19^ Department of Cardiothoracic Anaesthesiology, The Heart Center Copenhagen University Hospital—Rigshospitalet Copenhagen Denmark; ^20^ Department of Anesthesiology and Intensive Care Copenhagen University Hospital—Hvidovre Hvidovre Denmark; ^21^ Department of Anaesthesia and Intensive Care Copenhagen University Hospital—Bispebjerg and Frederiksberg Copenhagen Denmark

**Keywords:** critical care, empirical antibiotics, feasibility, intensive care, randomised clinical trial, sepsis

## Abstract

**Background:**

Meropenem and piperacillin/tazobactam are commonly used empirical antibiotics in critically ill adults with sepsis, but whether one is superior to the other is uncertain.

**Methods:**

The *Empirical Meropenem* versus *Piperacillin/Tazobactam for Adult Patients with Sepsis (EMPRESS)* trial is an ongoing investigator‐initiated, randomised, open‐label, adaptive clinical trial with an integrated feasibility phase comparing empirical treatment with meropenem versus piperacillin/tazobactam in critically ill adults with sepsis. The integrated feasibility phase enrolled 200 participants across 10 intensive care units (ICUs) in Denmark between 28 June and 12 December 2025. Five pre‐specified feasibility criteria were evaluated; if all feasibility criteria were met, the trial would proceed unaltered, whereas failure to meet one or more criteria would require intervention and re‐evaluation.

**Results:**

We randomised 200 of 284 screened patients (70.4%). The median age was 70 years (interquartile range (IQR): 60–77), 65.5% were males. At randomisation, 80.0% received vasopressors or inotropes, and 43.5% were on invasive mechanical ventilation. Four of five pre‐specified feasibility criteria were met: time to completion of the feasibility phase (5.5 months vs. threshold < 12.0 months), recruitment proportion (70.4% vs. threshold ≥ 50.0%), proportion of participants without consent to the continued collection of data (2.5% vs. threshold < 5.0%) and protocol adherence (81.0% vs. threshold ≥ 75.0%). The proportion of participants with timely primary outcome data availability (30‐day mortality) within 45 days was 85.5% and below the pre‐specified threshold of ≥ 95.0%. The proportions were low in the first 3 months (33.3%, 22.2% and 30.8%, respectively), increasing to 95.8% in the last month of the feasibility phase. All‐cause mortality at 30 days was 30.5%, and specific serious adverse reactions occurred in 4.0% of participants.

**Conclusions:**

In this integrated feasibility evaluation of the *EMPRESS* trial comparing empirical meropenem versus piperacillin/tazobactam in critically ill adults with sepsis, four of five pre‐specified feasibility criteria were met. The unmet criterion, timely primary outcome data availability, improved substantially during the feasibility phase. We consider the trial feasible and will proceed without modifications.

**Editorial Comment:**

This feasibility study assessed recruitment, randomised allocation and data collection for the multicentre EMPRESS trial. For adaptive trials on trial platforms, careful interim checking of trial design functions is an important and necessary process.

**Trial Registration:** Clinical Trials Information System EUCT number: 2023‐509703‐33‐00; ClinicalTrials.gov identifier: NCT06184659; Universal Trial Number: U1111‐1301‐6379

## 1 Introduction

Early administration of broad‐spectrum antibiotics is a cornerstone of sepsis management, and piperacillin/tazobactam and meropenem are among the most frequently used empirical antibiotic agents in the intensive care unit (ICU) [1, 2]. Carbapenems are often reserved as second‐ or third‐line agents to preserve their efficacy and reduce selection pressure for resistant organisms, a growing global health burden [3, 4].

Whether carbapenems are superior to piperacillin/tazobactam in critically ill adult patients with sepsis remains uncertain. A non‐inferiority randomised clinical trial (RCT) comparing definitive treatment with piperacillin/tazobactam versus meropenem in non‐critically ill patients with ceftriaxone‐resistant bacteraemia reported higher 30‐day mortality with piperacillin/tazobactam (12.3% vs. 3.7%; absolute risk difference 8.6% [1‐sided 97.5% confidence interval, CI: –∞ to 14.5%]) [5]. A systematic review and meta‐analysis similarly suggested worse outcomes, although statistically insignificant, with piperacillin/tazobactam compared with carbapenems (including a relative risk for all‐cause short‐term mortality of 1.16 [95% CI: 0.94–1.43]), though the certainty of evidence was low to very low and no trials were conducted exclusively in critically ill patients [6]. An international retrospective cohort study of ICU patients further supported these findings, though confounding could not be excluded [7]. Collectively, this indicates clinical equipoise and supports the need for a definitive RCT in critically ill adult patients with sepsis.

We designed the *Empirical Meropenem* versus *Piperacillin/Tazobactam for Adult Patients with Sepsis (EMPRESS)* trial as a randomised, open‐label, adaptive clinical trial, with an integrated feasibility phase [8]. An integrated feasibility phase was included to prospectively evaluate the operational viability of the adaptive trial design, the recruitment potential and the timely primary outcome data availability required for the planned adaptive analyses, before proceeding to full enrolment.

Here, we report the results of the integrated feasibility phase, including the first 200 participants.

## 2 Methods

### 2.1 Trial Design

*EMPRESS* is an investigator‐initiated, parallel‐group, randomised, open‐label, adaptive clinical trial with an integrated feasibility phase; the full design, rationale and analysis plan was published prior to the first enrolment [8]. *EMPRESS* is conducted in accordance with the *Declaration of Helsinki* [9] and *the International Council for Harmonisation Good Clinical Practice* guidelines [10]. *EMPRESS* was prospectively registered (EUCT number: 2023‐509703‐33‐00; ClinicalTrials.gov identifier: NCT06184659; Universal Trial Number: U1111‐1301‐6379). Reporting follows the *Consolidated Standards of Reporting Trials* (*CONSORT*) 2010 extension to randomised pilot and feasibility trials (Supporting Information) [11]. Enrolment continued uninterrupted during feasibility evaluation.

### 2.2 Study Setting

Enrolment in *EMPRESS* during the feasibility phase was conducted through the *Collaboration for Research in Intensive Care* (*CRIC*, www.cric.nu/empress) network at 10 participating ICUs, across nine Danish hospitals in all five Danish regions. The ICUs were recruited sequentially from June to December 2025; screening started on 12 June 2025 with the first participant randomised on 28 June 2025 (Table S1). Enrolment for the feasibility phase was completed on 12 December 2025, with follow‐up of data collection for the feasibility phase concluding on 26 January 2026.

### 2.3 Inclusion Criteria

Eligible patients were adults (≥ 18 years) presenting with critical illness and sepsis, including septic shock, with a clinical indication for empirical treatment with either meropenem or piperacillin/tazobactam [8]. Sepsis was defined in accordance with the *Sepsis‐3* criteria as suspected or documented infection combined with an acute increase of ≥ 2 points in the *Sequential Organ Failure Assessment* (*SOFA*) score [12]. Acute critical illness was defined as the requirement for invasive mechanical ventilation, non‐invasive ventilation, continuous use of continuous positive airway pressure (CPAP) for hypoxia, oxygen supplementation with flow ≥ 10 L/min or continuous infusion of any vasopressor or inotrope [8].

### 2.4 Exclusion Criteria

Patients were excluded for the following reasons: preceding intravenous (IV) treatment with meropenem or piperacillin/tazobactam for more than 24 h immediately prior to screening, suspected or confirmed pregnancy, known hypersensitivity to beta‐lactam antibiotics, suspected or documented central nervous system infection, known colonisation with bacteria resistant to either trial drug within 3 months, ongoing or planned treatment with valproate, informed consent following inclusion expected to be unobtainable or other criteria detailed in the published protocol [8].

### 2.5 Randomisation and Interventions

Randomisation was performed using a centralised, secure, web‐based screening, randomisation and electronic case report form (eCRF) system (*EasyRF*, www.easyrf.dk). During the feasibility phase, randomisation was stratified by trial site using computer‐generated allocation sequences with 1:1 allocation ratios and randomly varying block sizes; allocation sequences were concealed, with allocation revealed immediately after randomisation. Participants were randomly allocated to empirical IV treatment with either meropenem or piperacillin/tazobactam for up to 30 days after randomisation, or until discharge from the participating site, death, termination of empirical therapy (including initiation of definitive antibiotic treatment), withdrawal or change to a narrower‐spectrum empirical antibiotic in situations where no bacteria were identified on microbiological findings. Suggested doses were meropenem 1 g IV three times daily and piperacillin/tazobactam 4/0.5 g IV four times daily, administered as intermittent or prolonged (extended or continuous) infusions. All dosing decisions and adjustments were at the discretion of the treating clinician in accordance with local practice and guidelines, as were all co‐interventions, including combination antibiotic therapy. Blinding was not performed.

### 2.6 Feasibility Outcomes

Five pre‐specified feasibility criteria were evaluated after the first 200 participants had completed 30‐day follow‐up: (1) time to completion of the feasibility phase (feasibility threshold < 12 months); (2) recruitment proportion (the proportion of patients fulfilling the inclusion criteria, screened and subsequently randomised; threshold ≥ 50%); (3) proportion of participants without consent to the continued collection of data (threshold < 5%); (4) protocol adherence (defined as the proportion of participants without one or more protocol violations, see Section 2.8 for definitions; threshold: ≥ 75%) and (5) timely primary outcome data availability (proportion of participants with available primary outcome data (30‐day mortality) within 45 days, referred to as *retention proportion* in the published protocol [8]; threshold ≥ 95%), see Table 3 for full feasibility outcome definitions [8]. The 45‐day window reflects the 30‐day follow‐up period plus 15 additional days for data collection, as required for the adaptive analyses that will be conducted in the trial [8]. If all feasibility criteria were fulfilled, the trial was to proceed without modification. If one or more criteria were not fulfilled, corrective measures (e.g., additional site training) were to be done, the protocol was to be modified accordingly, both followed by re‐evaluation of feasibility or the trial stopped if deemed not feasible [8].

### 2.7 Clinical Outcomes

This evaluation includes all 30‐day clinical outcomes collected in the trial: all‐cause mortality (primary outcome, guiding all future adaptations in the trial), and one or more specific serious adverse reactions (SARs) (anaphylactic shock to IV meropenem or piperacillin/tazobactam, invasive fungal infection, pseudomembranous colitis or toxic epidermal necrolysis), new isolation precautions due to resistant bacteria, days alive without life support (invasive mechanical ventilation, circulatory support and renal replacement therapy, including days in‐between intermittent renal replacement therapy) and days alive out of hospital (secondary outcomes). For the *days alive* outcomes, non‐survivors were assigned 0 days, treating death as the worst possible outcome [13].

### 2.8 Data Collection and Follow‐Up

Participants were followed daily during hospital stay, with baseline characteristics and outcome data entered into the eCRF. Process variables related to protocol adherence, as well as the use of any other antibiotic agents, were registered within the first 30 days after randomisation. Major protocol violations were defined as the use of empirical antibiotic treatment regimens not including the allocated intervention, receipt of the other trial intervention on a non‐definitive indication or the administration of less than 50% of the prescribed daily dose of trial medication on any given day of the intervention period [8]. At 30 days, clinical staff reviewed participants' electronic patient records to ascertain vital status and confirm that all new admissions and new events had been correctly registered in the eCRF.

### 2.9 Statistical Analysis

Baseline, treatment characteristics and clinical outcomes are summarised descriptively, across all randomised participants without separation by allocation group, as medians with interquartile ranges (IQRs) for numerical variables and counts and percentages for categorical variables. Only the unblinded statistical team (not involved in recruitment, consent obtainment, treatment of participants, or outcome assessment) and the independent data monitoring and safety committee (IDMSC) are allowed to see outcome data separately by group [14].

Feasibility criteria were evaluated using descriptive statistics and assessed according to the pre‐specified thresholds. Time to completion of the feasibility phase and recruitment proportion were reported overall. Proportion of participants without consent to further data collection, timely primary outcome data availability and protocol adherence were reported both in total and stratified by treatment group. The feasibility criteria were evaluated against the total proportions only [8].

### 2.10 Sample Size

The sample size for the feasibility phase was determined based on practical considerations [15], sample sizes in other pilot/feasibility trials [16] and the expected precision of the feasibility outcome estimates [17]. The maximum sample size of *EMPRESS* is 14,000 participants. Across the evaluated scenarios, with varying assumed between‐arm differences, the expected sample size ranged from 2570 to 5859 participants [8].

## 3 Results

### 3.1 Participants

**FIGURE 1:**
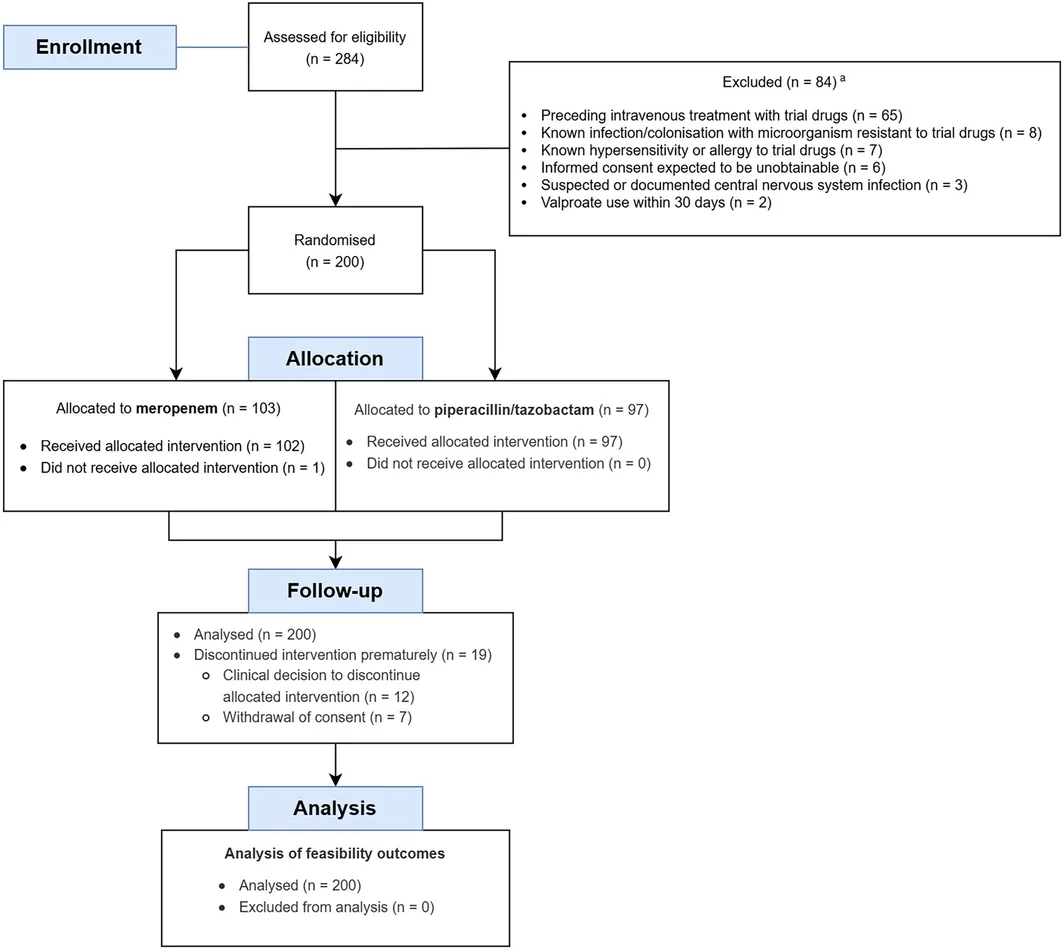
*CONsolidated Standards Of Reporting Trials* (*CONSORT*) flow diagram describing eligibility, screening and randomisation in the feasibility phase of the *EMPRESS* trial. The Follow‐Up and Analysis sections of the *CONSORT* flow diagram have been merged, as separation by allocation group is not presented during this reporting of the feasibility phase to prevent premature disclosure of outcome data that could compromise trial integrity prior to the pre‐specified final analysis. See the *EMPRESS* protocol for the full definitions of the exclusion criteria. ^a^Full definition of exclusion criteria can be found in the published protocol [8]. Patients could meet more than one exclusion criterion, so the reasons sum to more than the numbers excluded. EMPRESS: *Empirical Meropenem versus Piperacillin/Tazobactam for Adult Patients with Sepsis*; *n*, number.

In total, 200 of 284 patients screened for eligibility (70.4%) were randomised; 103 to meropenem and 97 to piperacillin/tazobactam (Figure 1). The most common reason for exclusion was preceding IV treatment with meropenem or piperacillin/tazobactam for more than 24 h prior to screening. All 200 randomised participants were included in the feasibility analyses.

### 3.2 Baseline Characteristics

Among enrolled participants, the median age was 70 years (IQR: 60–77), 65.5% were male and the median *Simplified Mortality Score for the Intensive Care Unit* (*SMS‐ICU*) [18] was 22 (IQR: 18–25), corresponding to an expected mortality at 90 days of approximately 40% (IQR: 28%–50%) [18]. At randomisation, 80.0% received vasopressors or inotropes, and 43.5% were on invasive mechanical ventilation (61.7% of those receiving respiratory support) (Table 1). The most common suspected primary sites of infection were pulmonary (50.0%) and abdominal (19.5%). The most frequently used antibiotics in the 24 h preceding randomisation were beta‐lactam/beta‐lactamase inhibitors (49.0%), nitroimidazoles (17.5%) and penicillins (15.0%). In 32.2% of participants, baseline cultures later returned with positive microbiological findings (details are presented in Table S2).

**TABLE 1:** Baseline characteristics among the 200 randomised participants.

Characteristic	Total (*N* = 200)
Age, median (IQR) (years)	70 (60–77)
Sex	
Female	69 (34.5%)
Male	131 (65.5%)
Weight, median (IQR) (kg)	80 (68–97)
Height, median (IQR) (cm)	174 (166–180)
Coexisting conditions	
Ischemic heart disease or heart failure	38 (19.0%)
Diabetes mellitus	61 (30.5%)
Chronic pulmonary disease	53 (26.6%)
Known immunosuppression	34 (17.0%)
Haematological or metastatic cancer^a^	23 (11.5%)
Known use of immunosuppressive therapy within the last 3 months^b^	20 (10.0%)
Previous organ transplantation	4 (2.0%)
Chronic use of renal replacement therapy^c^	8 (4.0%)
Chronic liver disease	7 (3.5%)
Clinical Frailty Scale, median (IQR)^d^	4.0 (3.0–5.0)
Limitations of care^e^	33 (16.5%)
Time from hospitalisation to enrolment, median (IQR) (days)	1.0 (1.0–3.0)
SMS‐ICU, median (IQR)^f^	22 (18–25)
Place of enrolment	
Intensive care unit	193 (96.5%)
Intermediate care unit	4 (2.0%)
Emergency department	2 (1.0%)
Hospital ward	1 (0.5%)
Primary site of infection^g^	
Pulmonary	100 (50.0%)
Gastrointestinal	39 (19.5%)
Urinary tract	32 (16.0%)
Skin or soft tissue	15 (7.5%)
Bloodstream	5 (2.5%)
Other	5 (2.5%)
Acute surgical admission^h^	54 (27.0%)
Oxygen supplementation^i^	
Invasive mechanical ventilation	87 (61.7%)
FiO_2_, median (IQR) (%)	50 (40–70)
Non‐invasive ventilation or continuous CPAP	28 (19.9%)
FiO_2_, median (IQR) (%)	58 (40–80)
Open system (≥ 10 L)	26 (18.4%)
O_2_‐flow, median (IQR) (L/min)	42 (20–50)
PaO_2_, median (IQR) (mmHg)^j^	78 (68–98)
SaO_2_, median (IQR) (%)	95 (92–97)
Lowest systolic blood pressure, median (IQR) (mmHg)	84 (71–99)
Highest lactate, median (IQR) (mmol/L)	2.7 (1.4–4.8)
Highest creatinine, median (IQR) (μmol/L)	133 (88–192)
Co‐interventions used at randomisation	
Systemic corticosteroids	68 (34.2%)
Vasopressors or inotropes^k^	160 (80.0%)
Renal replacement therapy	14 (7.0%)
Anti‐bacterial agents prior to randomisation^l^	
Beta‐lactam/beta‐lactamase inhibitor (e.g., piperacillin/tazobactam)	98 (49.0%)
Nitroimidazole (e.g., metronidazole)	35 (17.5%)
Penicillins (e.g., ampicillin, benzylpenicillin, dicloxacillin)	30 (15.0%)
Cephalosporin (e.g., cefuroxime, ceftriaxone)	17 (8.5%)
Aminoglycoside (e.g., gentamycin)	15 (7.5%)
Macrolide (e.g., clarithromycin)	14 (7.0%)
Carbapenem (e.g., meropenem)	5 (2.5%)
Fluoroquinolone (e.g., ciprofloxacin)	4 (2.0%)
Glycopeptide (e.g., vancomycin)	2 (1.0%)
Sulfonamide (e.g., sulfamethizole)	2 (1.0%)
Lincosamides (e.g., clindamycin)	1 (1.0%)
Other: any other antibiotic agent not included above	1 (0.5%)
One or more positive bacterial findings^m^	64 (32.2%)

*Note:* Values are presented as median (IQR) for continuous variables and counts (%) for categorical variables. All characteristics were recorded at the time of randomisation unless otherwise specified. The variable order and presentation follow the pre‐specified mock baseline table in the published *EMPRESS* trial protocol [8], data are presented in aggregate across allocation groups. One additional variable not included in the mock table has been added: one or more positive bacterial findings at baseline. Variables with any missing values were Clinical Frailty Scale (*n* = 6, 3.0%), primary site of infection (*n* = 4, 2.0%), height (*n* = 2, 1.0%), highest lactate (*n* = 2, 1.0%), weight (*n* = 1, 0.5%), highest creatinine (*n* = 1, 0.5%), chronic pulmonary disease (*n* = 1, 0.5%), systemic corticosteroids (*n* = 1, 0.5%) and positive bacterial findings (*n* = 1, 0.5%). All other variables had complete data.

Abbreviations: CPAP, continuous positive airway pressure; FiO_2_, fraction of inspired oxygen; ICU, intensive care unit; IQR, interquartile range; IV, intravenous; *n*, number; RRT, renal replacement therapy; SMS‐ICU, Simplified Mortality Score for the Intensive Care Unit.

^a^ Includes active haematological malignancy or known metastatic solid cancer at randomisation.

^b^ Defined as known haematological malignancy, use of immunosuppressive therapy within the preceding 3 months or prior solid organ transplantation.

^c^ Defined as chronic use of any form of renal replacement therapy (e.g., haemodialysis or peritoneal dialysis) at least once per week prior to the index hospital admission.

^d^ Clinical Frailty Scale, version 2.0 (range 1–9); investigator‐assessed at the time of randomisation based on information from the participant, relatives, and the electronic patient record. A score of 1 indicates very fit and 9 indicates terminally ill [19, 20].

^e^ Participant with limitation(s) in the use of life support (i.e., invasive mechanical ventilation, circulatory support, renal replacement therapy) and/or cardiopulmonary resuscitation at the time of randomisation.

^f^ *Simplified Mortality Score for the Intensive Care Unit* (*SMS‐ICU*); ranges from 0 to 42, with higher scores indicating greater mortality risk [18]. A median score of 22 corresponds to a predicted 90‐day mortality of approximately 40% in the original development and validation cohort (the IQR corresponds to approximately 28% to 50%), indicative of a severely ill population; absolute predicted mortalities should be interpreted with caution as calibration of prediction models deteriorates over time.

^g^ Based on clinical suspicion or documentation available at the time of randomisation.

^h^ Unplanned admission for an acute surgical diagnosis that initiated the current hospital stay.

^i^ Defined by the type of respiratory support ongoing at randomisation. Denominators reflect the number of participants receiving each type of support; participants receiving no respiratory support at randomisation are not included in these subgroups.

^j^ Corresponding to a median 10 (IQR: 9–13) kPa.

^k^ Defined as any continuous IV infusion of a vasopressor (e.g., norepinephrine, epinephrine, vasopressin or dopamine) or inotrope (e.g., dobutamine or milrinone) at the time of randomisation.

^l^ Any antibacterial agent (IV, oral or per gastrointestinal tube) commenced due to suspected or confirmed infection during the current hospital admission within 24 h prior to randomisation. Participants could receive more than one agent, so percentages sum to more than 100%.

^m^ Positive bacterial findings from samples drawn between 48 h before and 1 h after randomisation. Culture results were registered retrospectively when microbiological results were returned.

### 3.3 Treatment Characteristics

The allocated intervention was administered for a median of 4.0 days (IQR: 3.0–8.0), with intermittent infusion used in 53.5% and continuous infusion in 46.5% (Table 2). Empirical combination therapy was used in 40.5%, most commonly with a nitroimidazole (22%) or macrolide (13%).

**TABLE 2:** Treatment characteristics among the 200 randomised participants.

Treatment characteristics	Total (*n* = 200)
Dosing regimen^a^	
Continuous infusion	92 (46.5%)
Intermittent infusion	107 (53.5%)
Duration of allocated treatment regimen, median (IQR) (days)^b^	4.0 (3.0 to 8.0)
Reason for discontinuation of intervention	
Discharged or transferred to non‐participating site or out of hospital^c^	91 (45.5%)
Empirical or definitive treatment no longer indicated^d^	28 (14.0%)
Change to definitive antibiotic treatment	24 (12.0%)
Death	21 (10.5%)
Clinical decision to discontinue allocated intervention^e^	12 (6.0%)
Withdrawal of consent	7 (3.5%)
Clinical deterioration^f^	5 (2.5%)
Adverse reaction^g^	2 (1.0%)
De‐escalation to narrower antibiotic(s) during the empirical phase^h^	1 (0.5%)
Other reasons	9 (4.5%)
Concurrent empirical therapy during allocated empirical treatment (empirical combination therapy)	81 (40.5%)
Nitroimidazole	44 (22.0%)
Macrolide	26 (13.0%)
Glycopeptide	13 (6.5%)
Fluoroquinolone	6 (3.0%)
β‐lactam/β‐lactamase inhibitor	6 (3.0%)
Other(s)	5 (2.5%)
Oxazolidinone	3 (1.5%)
Penicillin	3 (1.5%)
Aminoglycoside	2 (1.0%)
Carbapenem	2 (1.0%)
Cephalosporin	1 (0.5%)
Sulphonamide	1 (0.5%)
Change from allocated empirical treatment strategy to other non‐trial empirical treatment strategy	11 (5.5%)
Clinical deterioration with indication for different antibiotic^f^	7 (3.5%)
De‐escalation to narrower empirical antibiotic(s) during the empirical phase^e^	1 (0.5%)
Change from allocated empirical treatment to other agent due to adverse reaction(s)^g^	1 (0.5%)
Other reason(s)	2 (1.0%)
Switch to definitive treatment^i^	33 (16.5%)
Penicillin	14 (7.0%)
Cephalosporin	8 (4.0%)
Carbapenem	6 (3.0%)
Fluoroquinolone	5 (2.5%)
β‐lactam/β‐lactamase inhibitor	5 (2.5%)
Nitroimidazole	3 (1.5%)
Glycopeptide	2 (1.0%)
Macrolide	2 (1.0%)
Aminoglycoside	1 (0.5%)
Other(s)	2 (1.0%)
Allocated antibiotic continued as definitive treatment: β‐lactam/β‐lactamase inhibitor	5 (2.5%)
Allocated antibiotic continued as definitive treatment: carbapenem	3 (1.5%)

*Note:* Values are presented as medians (IQRs) for continuous variables and counts (%) for categorical variables. There were no missing values except the daily question on whether the participant received at least 50% of the prescribed dose of trial medication, which had 0.2% missing values across all patient‐days (3 of 1667 daily observations), corresponding to missing observations in 1 participant (0.5%). The variables in this table derived from this daily question include duration of allocated treatment regimen, reason for discontinuation of intervention, reason for change in allocated empirical treatment strategy and switch to definitive treatment.

Abbreviations: IQR, interquartile range; *n*, number.

^a^ Dosing regimen of the first dose of the allocated study drug administered after randomisation, not including any loading dose.

^b^ Time from first to last administration of the allocated trial drug, in days.

^c^ Includes participants discharged to a non‐participating hospital department (89, 44.5%) or discharged out of hospital (2, 1.0%).

^d^ Including switch to prophylactic antibiotic treatment.

^e^ The clinician in conjunction with the site investigator (involving the coordinating investigator and sponsor if necessary) decide it is in the interest of the participant (e.g., due to potential interactions with other drugs that cannot be handled by dose adjustment).

^f^ Clinical deterioration with indication for a different antibiotic than allocated according to the treating clinician.

^g^ Adverse reaction to the allocated agent requiring a shift to a different antibiotic according to the treating clinician.

^h^ De‐escalation to another empirical antibiotic with narrower spectrum due to clinical improvement, not including the two trial interventions, in situations where no bacteria were found in microbiological findings.

^i^ Participants could receive more than one definitive agent, so the individual agents sum to more than the number of participants switching. Percentages are of all 200 randomised participants.

### 3.4 Feasibility Outcomes

Four of five pre‐specified feasibility criteria were met (Table 3). The unmet criterion was timely primary outcome data availability, which was available in 85.5% (threshold of ≥ 95%). Further exploration revealed a temporal pattern: monthly timely primary outcome data availability was low in the first 3 months (33.3%, 22.2% and 30.8%, respectively) but improved over time, with the criterion consistently met from October 2025 onwards, reaching 95.8% in the final month (Figures S3.1 and S3.2).

**TABLE 3:** Feasibility outcomes among the first 200 randomised participants in the *EMPRESS* trial.

Outcome	Total (*N* = 200)		Feasibility criteria^f^	Conclusions and actions
	Meropenem (*n* = 103)	Piperacillin/tazobactam (*n* = 97)		
Time to completion of feasibility phase (months)^a^	5.5 months		< 12 months	Met; proceeds without modification.
Recruitment proportion^b^	70.4%		≥ 50%	Met; proceeds without modification.
Proportion of participants without consent to the continued collection of data^c^	2.5%		< 5%	Met; proceeds without modification.
	2.9%	2.1%		
Timely primary outcome data availability^d^	85.5%		≥ 95%	Not met; monthly site reminders and eCRF data prompts implemented. Primary outcome data availability monitored continuously at each adaptive interim analysis and annual safety review.
	82.5%	88.7%		
Protocol adherence^e^	81.0%		≥ 75%	Met; proceeds without modification.

*Note:* Values are reported as months or percentages. Feasibility criteria were evaluated against pre‐specified thresholds after the first 200 participants completed 30‐day follow‐up. Proportion of participants without consent to the continued collection of data, timely primary outcome data availability and protocol adherence are reported both overall and stratified by allocated treatment group; feasibility criteria were evaluated against the aggregated proportions only. There were—by definition—no missing data for any feasibility outcomes.

Abbreviation: *EMPRESS*, *Empirical Meropenem versus Piperacillin/Tazobactam for Adult Patients with Sepsis*.

^a^ Time from randomisation of the first to randomisation of the 200th participant, in months.

^b^ Number of randomised participants divided by the total number of screened participants.

^c^ Number of participants for whom the consenting parties did not consent to the continued collection of data divided by the total number of randomised participants.

^d^ Number of participants with data on the primary outcome (all‐cause mortality at 30 days) within a maximum of 15 days beyond the scheduled follow‐up timepoint for each participant, divided by the total number of randomised participants. Referred to as *retention proportion* in the published protocol [8], but phrased differently here to avoid misinterpretation.

^e^ Number of participants without one or more protocol violations divided by the total number of randomised participants. A protocol violation was defined as (1) use of an empirical antibiotic regimen not including the allocated intervention; (2) receipt of the other trial intervention on a non‐definitive indication or (3) less than 50% of the prescribed daily dose of the allocated intervention administered on at least 1 day during the intervention period. De‐escalation to a narrower‐spectrum empirical antibiotic other than the two trial interventions, in the absence of positive findings, was not considered a protocol violation. Protocol adherence was evaluated systematically from the daily eCRF data, which captured if at least 50% of the prescribed daily dose of trial medication was administered and the use of any other antibiotic agents; the three protocol violation criteria were pre‐specified and applied to these data to derive the protocol adherence proportion.

^f^ Expected precision (95% confidence interval, Clopper–Pearson method) for each binary feasibility criterion at *n* = 200, assuming a proportion equal to the pre‐specified threshold: recruitment proportion ≥ 50%: 43%–57%; proportion of participants without consent to the continued collection of data < 5%: 2%–9%; protocol adherence ≥ 75%: 68%–81%; timely primary outcome data availability ≥ 95%: 91%–98% [8].

### 3.5 Clinical Outcomes

All‐cause mortality at 30 days was 30.5% (Table 4). One or more specific SARs within 30 days of randomisation occurred in 4.0% of participants. Invasive fungal infection and pseudomembranous colitis each occurred in 2.0% of participants; no cases of anaphylactic shock or toxic epidermal necrolysis to IV meropenem or piperacillin/tazobactam were observed. New isolation precautions due to resistant bacteria within 30 days occurred in 3.5%. Median days alive without life support at 30 days were 23 days (IQR: 0–27), and median days alive out of hospital at 30 days were 0 days (IQR: 0–18).

**TABLE 4:** Clinical outcomes among the 200 randomised participants.

Outcome	Total (*N* = 200)
All‐cause mortality at 30 days	61 (30.5%)
One or more specific serious adverse reactions (SARs) at 30 days^a^	8 (4.0%)
Invasive fungal infection	4 (2.0%)
Pseudomembranous colitis	4 (2.0%)
Anaphylactic shock due to IV piperacillin/tazobactam or meropenem	0 (0.0%)
Toxic epidermal necrolysis	0 (0.0%)
New isolation precaution due to one or more resistant bacteria at 30 days	7 (3.5%)
Days alive without life support at 30 days^b^	23.0 (0.0–27.0)
Days alive without invasive mechanical ventilation^b^	25.0 (0.0–30.0)
Days alive without circulatory support^b^	25.0 (0.0–28.0)
Days alive without renal replacement therapy^b^	30.0 (0.0–30.0)
Days alive out of hospital at 30 days^c^	0.0 (0.0–18.0)

*Note:* Values are presented as counts (%) for binary outcomes and median (IQR) for count outcomes. Clinical outcomes are reported descriptively across all participants without separation by allocation. See Supporting Information Figure S5 for proportions of participants in one of four mutually exclusive states (out of hospital, hospitalised without life support, on life support or dead) on a given day. There were no missing data for any of the clinical outcomes.

Abbreviations: IQR, interquartile range; IV, intravenous; SAR, serious adverse reaction.

^a^ Number of participants experiencing one or more of the following pre‐specified, specific serious adverse reactions within 30 days of randomisation: anaphylactic shock to IV piperacillin/tazobactam or meropenem, invasive fungal infection, pseudomembranous colitis or toxic epidermal necrolysis.

^b^ All participants who died during the follow‐up period were assigned 0 days alive without life support, treating death as the worst possible outcome. Life support is defined as invasive mechanical ventilation, circulatory support (vasopressors or inotropes) or renal replacement therapy; for renal replacement therapy, any gap of up to 3 days between two renal replacement therapy sessions is counted as days with renal replacement therapy.

^c^ All participants who died during the follow‐up period were assigned 0 days alive out of hospital, treating death as the worst possible outcome [13].

## 4 Discussion

In the integrated feasibility phase of the *EMPRESS* trial, four of five pre‐specified feasibility criteria were met. Enrolment of 200 participants was completed in 5.5 months across 10 Danish ICUs, with a recruitment proportion of 70.4%, protocol adherence of 81.0% and a proportion without consent to further data collection of 2.5%, all meeting the pre‐specified thresholds. The unmet criterion was timely primary outcome data availability of 85.5%, which was lower than expected.

There was a temporal pattern for timely primary outcome data availability: it was low in the first months, improved over time and was consistently over the 95% threshold from October 2025 onwards (Figures S3.1 and S3.2). This is likely explained by early operational challenges rather than a structural problem with the trial, a pattern previously observed in other feasibility trials where site‐level experience and protocol familiarity improved adherence over time [21]. Contributing factors included insufficiently established standard operating procedures for follow‐up data collection at the initial sites, the summer holiday period occurring during the first months of enrolment and early functionality issues in the eCRF that required some data to be re‐entered, and that queries for missing data were not activated until all 200 participants had been enrolled. Accordingly, monthly site reminders for unresolved follow‐up data and planned functionality upgrades within the eCRF have been implemented, and primary outcome data completeness will be monitored continuously at each adaptive analysis (after 400 participants and subsequently after every additional 300 participants) [8].

The observed all‐cause mortality at 30 days of 30.5% exceeded the 25% assumed in the planning of *EMPRESS* [8]. This may reflect case mix of the feasibility cohort, as the highly specialised university hospitals that opened first are likely to care for a higher‐acuity patient population than the mix of sites expected in the full trial (Supporting Information Table S4). The observed rate is furthermore consistent with the upper range of large international ICU sepsis cohort [22]. In sensitivity analyses of adaptive trial performance metrics performed during trial design using a 30% mortality, performance metrics were not substantially affected [8]. We will continuously monitor the mortality proportion throughout the main trial. Should it not stabilise within the evaluated event proportions assessed during the trial design phase, we will consider additional sensitivity analyses of the trial design performance. Specific SARs occurred in 4.0% of participants, with invasive fungal infection and pseudomembranous colitis each occurring in 2.0%; this is expected in critically ill adults with sepsis and raises no concerns [23, 24, 25].

Prior antibiotic use at randomisation was common, most frequently beta‐lactam/beta‐lactamase inhibitors in 49.0% of participants, consistent with previously observed antibiotic prescribing patterns in critically ill patients with sepsis [7, 22]. This prior exposure did not preclude eligibility in *EMPRESS* provided that prior exposure to either trial drug did not exceed 24 h immediately prior to screening, thereby preserving clinical equipoise around the empirical choice while capturing a population representative of everyday ICU practice. In a recent large biomarker‐guided antibiotic trial in sepsis, prior antibiotic exposure exceeding 24 h was the most common reason for exclusion, underscoring its prevalence in this setting [26]. Notably, positive bacterial findings were found in baseline cultures in 32.2% of participants; however, as microbiological results were pending at the time of randomisation, empirical treatment was initiated before culture results were available, which is typical of the clinical scenario *EMPRESS* is designed to address.

### 4.1 Strengths and Limitations

The *EMPRESS* feasibility phase has several strengths. The integrated feasibility phase allowed for the prospective evaluation of trial viability before proceeding to full international expansion, with pre‐specified criteria and decision rules defined a priori [8]. The pragmatic eligibility criteria and the use of deferred informed consent, as applicable under European clinical trial legislation, facilitated enrolment of a broad and representative population of critically ill adults with sepsis, including those most severely ill, as reflected by the 80% receiving vasopressors or inotropes at randomisation and an observed mortality of 30.5% at 30 days. Finally, enrolment across 10 Danish ICUs spanning all five Danish regions within 5.5 months demonstrates that the trial infrastructure and network can support enrolment required for a full‐size adaptive trial.

The *EMPRESS* feasibility phase has limitations too. Restriction to Danish ICUs limits the generalisability of these findings, however, the trial is planned to expand internationally. The assessed feasibility metrics will be continuously evaluated (in conjunction with the annual safety reporting, and at the time of each adaptive analysis for primary outcome data availability) [8], providing the possibility to adjust trial procedures and provide further training if needed. Timely primary outcome availability is particularly important because the adaptive (interim) analyses must be conducted without unnecessary delay. Although the proportion of participants with timely primary outcome data during the overall feasibility phase was below the predefined threshold, the substantial and consistent improvement observed from October 2025 onwards was reassuring. Primary outcome data availability will be evaluated at each adaptive analysis, that is, after 400 participants and every additional 300 participants [8]. Finally, as the screening log captures only patients formally screened in the eCRF, the recruitment proportion does not account for potentially eligible but unscreened patients and may overestimate the true proportion [21, 27].

## 5 Conclusions

In this integrated feasibility evaluation of the *EMPRESS* trial comparing empirical meropenem versus piperacillin/tazobactam in critically ill adults with sepsis, four of five pre‐specified criteria were met. The unmet criterion, timely primary outcome data availability, improved substantially over the feasibility phase, consistently meeting the 95% threshold from October 2025 onwards. We therefore consider the trial feasible and proceed without modification with primary outcome data availability continuously evaluated at each adaptive analysis.

## Author Contributions

**Morten Hylander Møller**, **Fredrik Sjövall**, **Marie Warrer Munch**, **Anders Perner** and **Anders Granholm:** conceptualisation. **Nick Frørup Meier**, **Morten Hylander Møller**, **Marie Warrer Munch**, **Frederik Boëtius Hertz**, **Marie Helleberg**, **Fredrik Sjövall**, **Anders Perner** and **Anders Granholm:** funding acquisition. **Nick Frørup Meier** and **Anders Granholm:** formal analysis. **Nick Frørup Meier**, **Tine Sylvest Meyhoff**, **Carl Thomas Anthon** and **Anders Granholm:** data curation. **Nick Frørup Meier**, **Morten Hylander Møller**, **Marie Warrer Munch**, **Fredrik Sjövall**, **Marie Helleberg**, **Frederik Boëtius Hertz**, **Benjamin Skov Kaas‐Hansen**, **Bodil Steen Rasmussen**, **Aksel Karl Georg Jensen**, **Theis Lange**, **Anders Perner** and **Anders Granholm:** methodology. **Nick Frørup Meier**, **Morten Hylander Møller**, **Maj‐Brit Nørregaard Kjær**, **Fredrik Sjövall**, **Anders Perner** and **Anders Granholm:** project administration. **Morten Hylander Møller**, **Maj‐Brit Nørregaard Kjær**, **Anders Perner** and **Anders Granholm:** supervision. **Nick Frørup Meier:** writing – original draft. All authors: investigation and writing – review and editing.

## Funding

The *EMPRESS* trial is funded by grants from the Independent Research Fund Denmark, the Research Council at Rigshospitalet, the Research Fund for Health Research of the Capital Region of Denmark, the Beckett Foundation, Læge Inger Goldmanns Fond, Grosserer Jakob Ehrenreich og Hustru Grete Ehrenreichs Fond and the Swedish Research Council. The trial uses infrastructure and methodology developed as part of the *Intensive Care Platform Trial* (*INCEPT*) programme [8] (www.incept.dk), which is funded by the Novo Nordisk Foundation and Sygeforsikringen ‘danmark’ and supported by Grosserer Jakob Ehrenreich og Hustru Grete Ehrenreichs Fond, Dagmar Marshalls Fond and Savværksejer Jeppe Juhl og hustru Ovita Juhls Mindelegat. The funding organisations have not been and will not be involved in the design, conduct, analyses or reporting of the trial nor will they have ownership of the data.

## Conflicts of Interest

The Department of Intensive Care at Copenhagen University Hospital—Rigshospitalet (N.F.M., M.H.M., M.‐B.N.K., M.W.M., B.S.K.‐H., H.‐C.T.‐M., A.K.G.J., J.S.A., D.P., T.S.M., C.T.A., A.T., M.G.‐D., Z.E., A.W.K., K.R.U., J.A.B., R.F.L., A.P. and A.G.) has received grants from the Novo Nordisk Foundation and Sygeforsikringen ‘danmark’ outside the submitted work. M.H. has participated in advisory boards for GlaxoSmithKline, AstraZeneca, Pfizer, Sobi, Roche and MSD. The other authors declare no conflicts of interest.

## Supporting information


**Table S1:** Enrolment by site during the EMPRESS feasibility phase.
**Table S2:** Positive baseline bacterial findings among the included participants.
**Supporting Information: S3** Timely primary outcome data availability over time in the *EMPRESS* feasibility phase.
**Figure S3.1** Monthly and cumulative timely primary outcome data availability proportions over time in the total cohort.
**Figure S3.2** Monthly and cumulative timely primary outcome data availability proportions over time, stratified by allocated treatment groups.
**Supporting Information: S4** Monthly enrolment and all‐cause mortality at 30 days during the *EMPRESS* feasibility phase.
**Supporting Information: S5** Daily participant states from randomisation to 30 days among the 200 randomised participants.
**Supporting Information:**  Completed CONSORT 2010 checklist of information to include when reporting a pilot or feasibility trial.

## Data Availability

The data that support the findings of this study are available from the corresponding author upon reasonable request, subject to approval by the EMPRESS management committee. De‐identified individual participant data (without personal, identifiable information, with timestamps replaced by relative time differences with respect to the time of randomisation and other measures as deemed relevant) may be shared following a research proposal outlining objectives, methodology and planned use, and after the necessary approvals. Alternatively, aggregated, scrambled or synthetic datasets may be shared. Data will generally be shared only after publication of the primary results of the EMPRESS trial, including a grace period of at least 9 months [8]. Approved researchers will sign appropriate agreements to ensure compliance with the approved purpose and with ethical and legal requirements.
