# Supplementary material for: Empirical Meropenem Versus Piperacillin/Tazobactam for Critically Ill Adults With Sepsis: Feasibility of a Randomised Trial
Source: Acta Anaesthesiol Scand. 2026 Aug 31;70(9):e70324. doi: 10.1111/aas.70324 (PMC13529373; doi:10.1111/aas.70324)
Supplement: Supplementary file 1 [file AAS-70-0-s001.pdf]

EMPIRICAL MEROPENEM VERSUS PIPERACILLIN/TAZOBACTAM FOR CRITICALLY ILL ADULTS WITH SEPSIS: FEASIBILITY OF A RANDOMISED TRIAL

AUTHORS

Nick Frørup Meier[1,2], Morten Hylander Møller[1,2,3], Maj-Brit Nørregaard Kjær[1,2], Fredrik Sjövall[4,5], Marie Warrer Munch[1,2], Benjamin Skov Kaas-Hansen[1,2], Hans-Christian Thorsen-Meyer[1,2], Bodil Steen Rasmussen[2,6,7], Marie Helleberg[8], Frederik Boëtius Hertz[9,10], Theis Lange[2,11], Aksel Karl Georg Jensen[1,11], Lars Wiuff Andersen[12,13,14], Jakob Steen Andersen[1], Jens Michelsen[15], Lars Peter Kloster Andersen[2,3,16], Morten Heiberg Bestle[2,3,17], Anne Craveiro Brøchner[2,18], Theis Skovsgaard Itenov[2,3,19], Steffen Christensen[12,13], Ronni Thermann Reitz Plovsing[20], Klaus Ulrik Koch[12], Anders Møller[21], Olav Lilleholt Schjørring[2,6,7], Naia Bech Cranø[16], Kasper Vamdrup-Andersen[15], Davide Placido[1,2], Tine Sylvest Meyhoff[1,2], Carl Thomas Anthon[1,2], Aske Tøgern[1,2], Milda Grigonyte-Daraskeviciene[1,2], Zemira Engbakken[1,2], Anne Witte Kamstrup[1,2], Kis Rønn Uhre[1,2], Jehad Ahmad Barakji[1,2], Rikke Faebo Larsen[1,2], Anders Perner[1,2,3], Anders Granholm[1,2,11]

AFFILIATIONS

[1] Department of Intensive Care, Copenhagen University Hospital — Rigshospitalet, Copenhagen, Denmark

[2] Collaboration for Research in Intensive Care (CRIC), Copenhagen, Denmark

[3] Department of Clinical Medicine, University of Copenhagen, Copenhagen, Denmark

[4] Department of Intensive and Perioperative Care, Skåne University Hospital, Malmö, Sweden

[5] Department of Clinical Sciences, Lund University, Malmö, Sweden

[6] Department of Anaesthesia and Intensive Care, Aalborg University Hospital, Aalborg, Denmark

[7] Department of Clinical Medicine, Aalborg University, Aalborg, Denmark

[8] Department of Infectious Diseases, Copenhagen University Hospital — Rigshospitalet, Copenhagen, Denmark

[9] Department of Clinical Microbiology, Copenhagen University Hospital — Rigshospitalet, Copenhagen, Denmark

[10] Department of Immunology and Microbiology, University of Copenhagen, Copenhagen, Denmark

[11] Section of Biostatistics, Department of Public Health, University of Copenhagen, Copenhagen, Denmark

[12] Department of Anesthesiology and Intensive Care Medicine, Aarhus University Hospital, Aarhus, Denmark

[13] Department of Clinical Medicine, Aarhus University, Aarhus, Denmark

[14] Prehospital Emergency Medical Services, Aarhus, Central Denmark Region, Denmark

[15] Department of Anaesthesiology and Intensive Care Medicine, Odense University Hospital, Odense, Denmark

[16] Department of Anaesthesiology, Zealand University Hospital, Køge, Denmark

[17] Department of Intensive Care, Copenhagen University Hospital — North Zealand, Hillerød, Denmark

[18] Department of Anaesthesiology and Intensive Care, Lillebælt Hospital, Kolding, Denmark

[19] Department of Cardiothoracic Anaesthesiology, The Heart Center, Copenhagen University Hospital—Rigshospitalet, Copenhagen, Denmark

[20] Department of Anesthesiology and Intensive Care, Copenhagen University Hospital-Hvidovre Hospital, Hvidovre, Denmark

[21] Department of Anaesthesia and Intensive Care, Copenhagen University Hospital — Bispebjerg and Frederiksberg, Copenhagen, Denmark

CONTENTS

This supplementary material provides additional methodological detail and results supporting the main manuscript. The full *EMPRESS* trial protocol, including the statistical analysis plan and any subsequent amendments, is available at www.cric.nu/empress. The supplementary material should be read in conjunction with the main manuscript and the published *EMPRESS* protocol paper.[1]

Table of contents

Table S1: Enrolment by site during the EMPRESS feasibility phase 4
Table S2: Positive baseline bacterial findings among the included participants 5
S3: Timely primary outcome data availability over time in the *EMPRESS* feasibility phase 7
Figure S3.1 Monthly and cumulative timely primary outcome data availability proportions over time in the total cohort 7
Figure S3.2 Monthly and cumulative timely primary outcome data availability proportions over time, stratified by allocated treatment groups 8
S4: Monthly enrolment and all-cause mortality at 30 days during the *EMPRESS* feasibility phase 9
S5: Daily participant states from randomisation to 30 days among the 200 randomised participants 10
Completed CONSORT 2010 checklist of information to include when reporting a pilot or feasibility trial .. 11
References 14

Table S1: Enrolment by site during the EMPRESS feasibility phase

Danish participating sites	Start date	Enrolled (n = 200)
Copenhagen University Hospital – Rigshospitalet ICU, 4131	12 June 2025	41 (20.5%)
Aalborg University Hospital ICU	26 June 2025	48 (24.0%)
Zealand University Hospital, Køge ICU	26 June 2025	34 (17.0%)
North Zealand University Hospital, Hillerød ICU	14 August 2025	10 (5.0%)
Odense University Hospital ICU	8 September 2025	33 (16.5%)
Lillebælt Hospital, Kolding ICU	8 September 2025	9 (4.5%)
Aarhus University Hospital ICU North	2 October 2025	2 (1%)
Aarhus University Hospital ICU East	8 October 2025	13 (6.5%)
Copenhagen University Hospital – Bispebjerg	5 December 2025	9 (4.5%)
Copenhagen University Hospital – Hvidovre	8 December 2025	1 (0.5%)

The table displays the number and proportion of participants enrolled at each of the 10 participating ICUs across Denmark between 28 June and 12 December 2025.

Abbreviations: ICU: intensive care unit.

Table S2: Positive baseline bacterial findings among the included participants

	**Total (n = 200)**
Positive bacterial findings in baseline samples [a]	64 (32.2%)
**Type of sample**	
Blood	42 (21.1%)
Lower airway secretions or bronchoalveolar lavage	18 (9.0%)
Tissue, bone, or pus	18 (9.0%)
Urine	17 (8.5%)
Peritoneal fluid	1 (0.5%)
**Type or bacteria**	
Escherichia coli	25 (12.6%)
Klebsiella spp.	13 (6.5%)
Other *Streptococcus*	8 (4.0%)
Methicillin-sensitive *Staphylococcus aureus* (MSSA)	7 (3.5%)
Streptococcus pneumoniae	6 (3.0%)
Enterobacter spp.	5 (2.5%)
Haemophilus influenza	5 (2.5%)
Enterococcus faecalis	3 (1.5%)
Enterococcus faecium	3 (1.5%)
Aeromonas spp.	2 (1.0%)
Aerococcus spp	1 (0.5%)
Methicillin-resistant *Staphylococcus aureus* (MRSA)	1 (0.5%)
Other *Enterococcus*	1 (0.5%)
Proteus spp.	1 (0.5%)
Pseudomonas spp	1 (0.5%)
Other bacteria	12 (6.0%)
**Isolate with resistance to at least one of the trial drugs**	
Resistance to either piperacillin/tazobactam or meropenem	4 (2.0%)
Resistance to piperacillin/tazobactam	2 (1.0%)
Resistance to meropenem	4 (2.0%)

Positive baseline findings include all reported isolates and were not adjudicated for clinical relevance (e.g., contaminants vs pathogens). Values are presented as counts (%) unless otherwise specified. Resistance is defined as at least one finding showing resistance to the

respective agent. Abbreviations: n: number; *spp*.: species. One participant had missing baseline microbiological data. Two participants had missing data for the type of bacteria.

a) Samples were drawn between 48 hours before and 1 hour after randomisation and registered retrospectively when results were available; a positive finding does therefore not imply that results were known at the time of randomisation. Among the 200 participants with data, 64 (32.2%) had at least one positive bacterial finding, with a total of 96 positive isolates identified across these 64 participants. As a participant could have had multiple positive samples with different organisms, the number of isolates and the sum of percentages for type of sample and type of bacteria exceed the number of participants with positive finding.

S3: Timely primary outcome data availability over time in the *EMPRESS* feasibility phase

Figure S3.1 Monthly and cumulative timely primary outcome data availability proportions over time in the total cohort

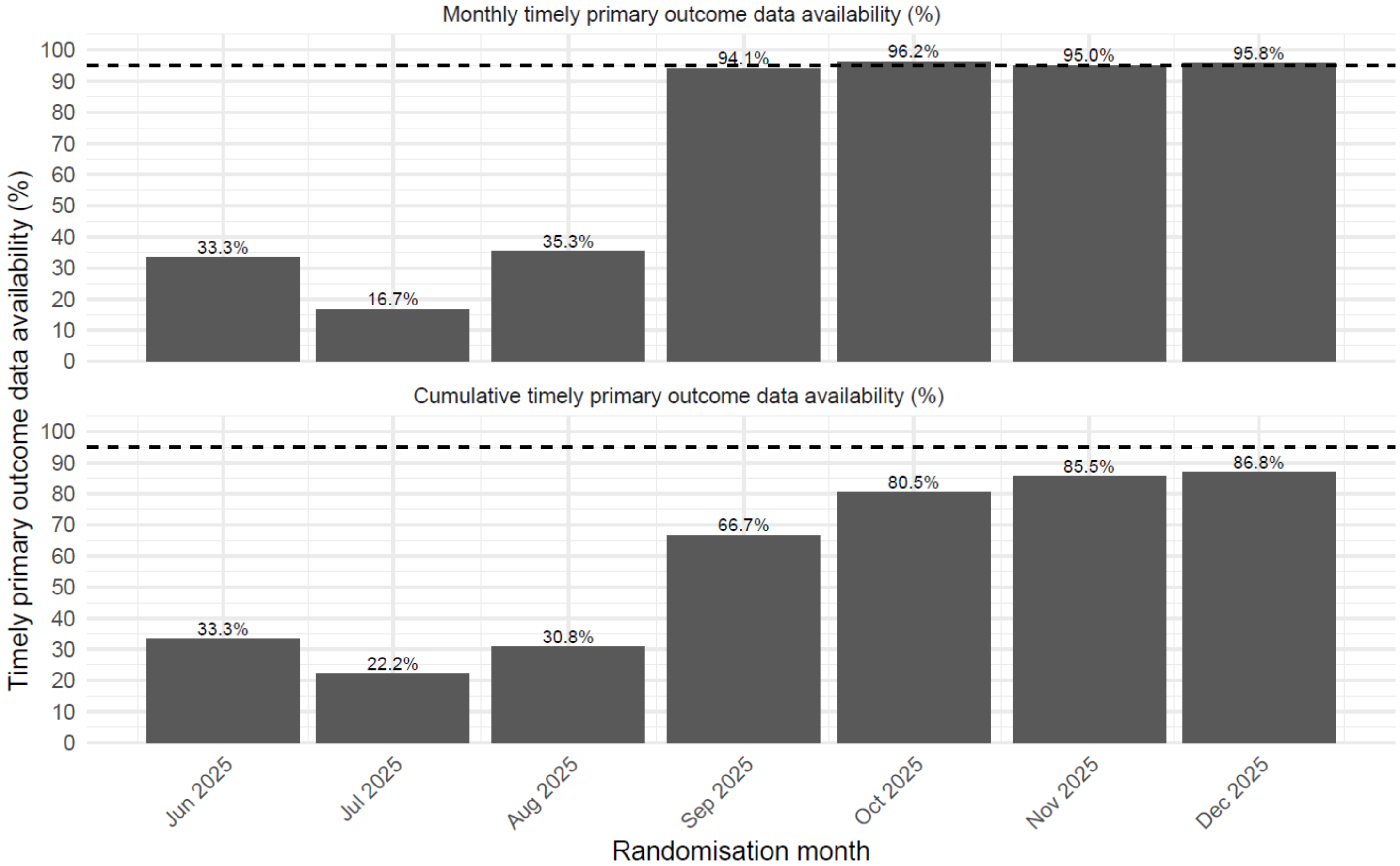

Each bar represents the monthly proportions of timely primary outcome data availability (y-axis) for participants randomised in each month, defined as the proportion with data on the primary outcome within a maximum of 15 days beyond the scheduled 30-day follow-up timepoint (referred to as retention in the protocol,[1] but rephrased here for clarity). The dashed horizontal line indicates the pre-specified timely outcome data availability threshold of 95%.

Figure S3.2 Monthly and cumulative timely primary outcome data availability proportions over time, stratified by allocated treatment groups

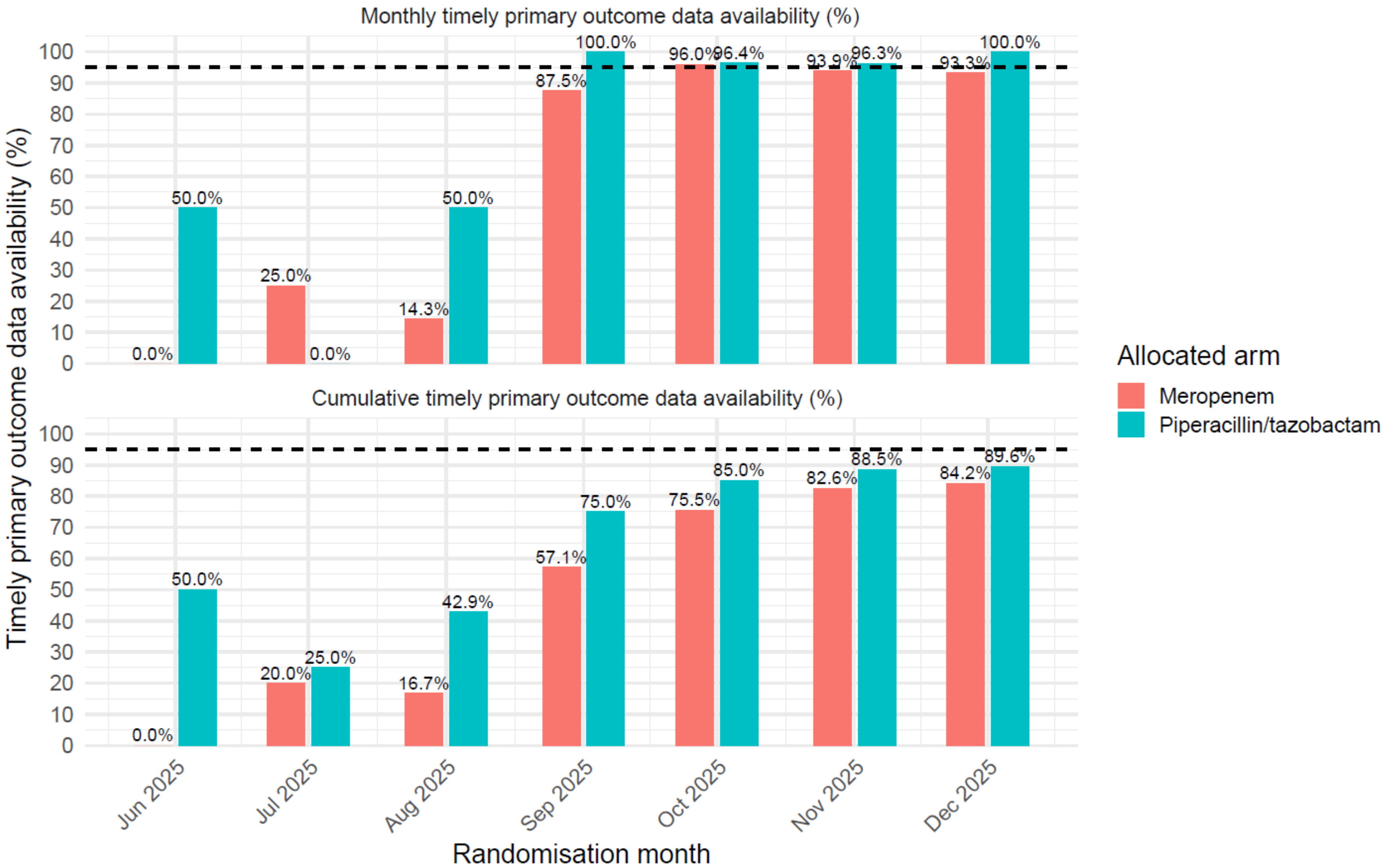

Each bar represents the monthly timely primary outcome data availability proportion (y-axis) for participants randomised in each month, shown separately for the meropenem (red) and piperacillin/tazobactam (light blue) groups. The dashed horizontal line indicates the pre-specified timely outcome data availability threshold of 95%.

S4: Monthly enrolment and all-cause mortality at 30 days during the *EMPRESS* feasibility phase

Month	Active sites, n	Participants enrolled, n	All-cause mortality at 30 days, n (%)
June 2025	3	3	1 (33.3%)
July 2025	3	6	1 (16.7%)
August 2025	4	17	5 (29.4%)
September 2025	6	36	7 (19.4%)
October 2025	8	53	21 (39.6%)
November 2025	8	61	20 (32.8%)
December 2025	10	24	6 (25.0%)
**Total**	**10**	**200**	**61 (30.5%)**

Values are counts (%) unless otherwise specified. The number of active sites reflects the number of ICUs that had commenced enrolment by the end of each calendar month. All-cause mortality at 30 days is reported per month of randomisation. Monthly denominators are small and the observed month-to-month variation in mortality should be interpreted with caution, as it is subject to considerable sampling variability.

Abbreviations: ICU: intensive care unit; n: number.

S5: Daily participant states from randomisation to 30 days among the 200 randomised participants

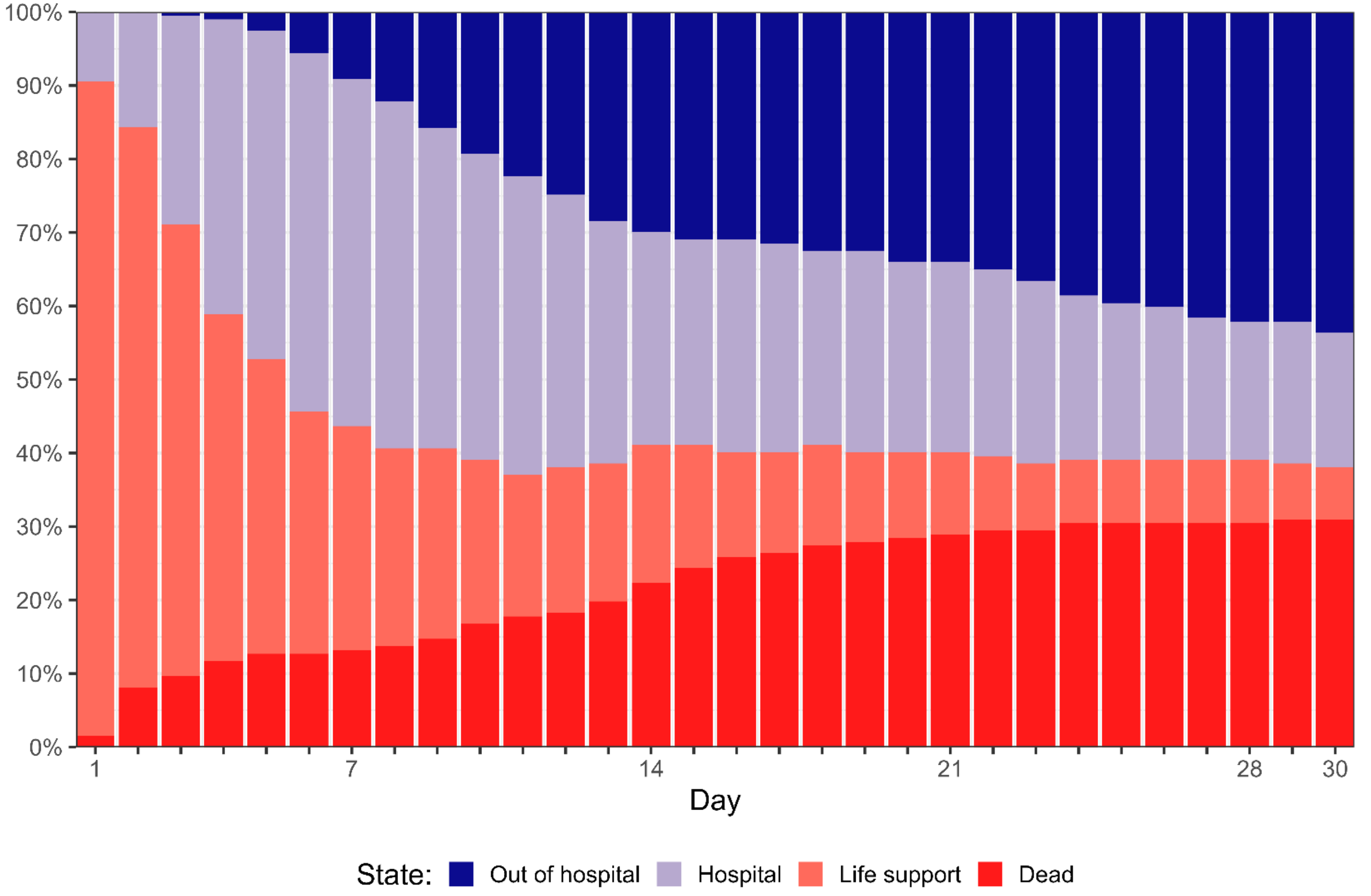

Each bar represents the proportion of participants in one of four mutually exclusive states on a given day: dead; receiving life support (invasive mechanical ventilation, circulatory support, or renal replacement therapy) in hospital; in hospital without life support; or out of hospital and alive. The horizontal axis shows days from randomisation. There were no missing data for the clinical outcomes.

Completed CONSORT 2010 checklist of information to include when reporting a pilot or feasibility trial

Completed CONSORT 2010 checklist for reporting pilot or feasibility trials;[2] edited to report section numbers instead of page numbers.

Section/Topic	Item No	Checklist item	Reported in section no.
**Title and abstract**			
	1a	Identification as a pilot or feasibility randomised trial in the title	Title page
	1b	Structured summary of pilot trial design, methods, results, and conclusions (for specific guidance see CONSORT abstract extension for pilot trials)	Abstract
**Introduction**			
Background and objectives	2a	Scientific background and explanation of rationale for future definitive trial, and reasons for randomised pilot trial	1 Introduction
	2b	Specific objectives or research questions for pilot trial	1 Introduction
**Methods**			
Trial design	3a	Description of pilot trial design (such as parallel, factorial) including allocation ratio	2.1 Trial design
	3b	Important changes to methods after pilot trial commencement (such as eligibility criteria), with reasons	2.1 Trial design
Participants	4a	Eligibility criteria for participants	2.3 Inclusion criteria; 2.4 Exclusion criteria
	4b	Settings and locations where the data were collected	2.2 Study setting
	4c	How participants were identified and consented	2.1 Trial design; 2.8 Data collection and follow-up details
Interventions	5	The interventions for each group with sufficient details to allow replication, including how and when they were actually administered	2.5 Randomisation and interventions
Outcomes	6a	Completely defined prespecified assessments or measurements to address each pilot trial objective specified in 2b, including how and when they were assessed	2.6 Feasibility outcomes; 2.7 Clinical outcomes
	6b	Any changes to pilot trial assessments or measurements after the pilot trial commenced, with reasons	2.1 Trial design

	6c	If applicable, prespecified criteria used to judge whether, or how, to proceed with future definitive trial	2.6 Feasibility outcomes; Table 3
Sample size	7a	Rationale for numbers in the pilot trial	2.10 Sample size
	7b	When applicable, explanation of any interim analyses and stopping guidelines	2.10 Sample size
Randomisation:			
Sequence generation	8a	Method used to generate the random allocation sequence	2.5 Randomisation and interventions
	8b	Type of randomisation(s); details of any restriction (such as blocking and block size)	2.5 Randomisation and interventions
Allocation concealment mechanism	9	Mechanism used to implement the random allocation sequence (such as sequentially numbered containers), describing any steps taken to conceal the sequence until interventions were assigned	2.5 Randomisation and interventions
Implementation	10	Who generated the random allocation sequence, who enrolled participants, and who assigned participants to interventions	2.5 Randomisation and interventions
Blinding	11a	If done, who was blinded after assignment to interventions (for example, participants, care providers, those assessing outcomes) and how	2.5 Randomisation and interventions
	11b	If relevant, description of the similarity of interventions	Not applicable (trial not blinded)
Statistical methods	12	Methods used to address each pilot trial objective whether qualitative or quantitative	2.9 Statistical analysis
**Results**			
Participant flow (a diagram is strongly recommended)	13a	For each group, the numbers of participants who were approached and/or assessed for eligibility, randomly assigned, received intended treatment, and were assessed for each objective	3.1 Participants; Figure 1
	13b	For each group, losses and exclusions after randomisation, together with reasons	3.1 Participants; Figure 1
Recruitment	14a	Dates defining the periods of recruitment and follow-up	2.2 Study setting
	14b	Why the pilot trial ended or was stopped	2.1 Trial design
Baseline data	15	A table showing baseline demographic and clinical characteristics for each group	3.2 Baseline characteristics; Table 1
Numbers analysed	16	For each objective, number of participants (denominator) included in each analysis. If relevant, these numbers should be by randomised group	3.1 Participants; Table 3
Outcomes and estimation	17	For each objective, results including expressions of uncertainty (such as 95% confidence interval) for any estimates. If relevant, these results should be by randomised group	3.4 Feasibility outcomes; 3.5 Clinical outcomes; Tables 3, 4; Supporting Information

Ancillary analyses	18	Results of any other analyses performed that could be used to inform the future definitive trial	3.4 Feasibility outcomes; 3.5 Clinical outcomes; Supporting Information (Figures S3.1–S3.2, Table S4)
Harms	19	All-important harms or unintended effects in each group (for specific guidance see CONSORT for harms)	3.5 Clinical outcomes; Table 4
	19a	If relevant, other important unintended consequences	3.5 Clinical outcomes
**Discussion**			
Limitations	20	Pilot trial limitations, addressing sources of potential bias and remaining uncertainty about feasibility	4.1 Strengths and limitations
Generalisability	21	Generalisability (applicability) of pilot trial methods and findings to future definitive trial and other studies	4.1 Strengths and limitations
Interpretation	22	Interpretation consistent with pilot trial objectives and findings, balancing potential benefits and harms, and considering other relevant evidence	4.0 Discussion; 5.0 Conclusion
	22a	Implications for progression from pilot to future definitive trial, including any proposed amendments	4.0 Discussion; 5.0 Conclusion
**Other information**			
Registration	23	Registration number for pilot trial and name of trial registry	Title page
Protocol	24	Where the pilot trial protocol can be accessed, if available	2.1 Trial design; www.cric.nu/empress
Funding	25	Sources of funding and other support (such as supply of drugs), role of funders	Funding and Financial support
	26	Ethical approval or approval by research review committee, confirmed with reference number	2.1 Trial design; 2.2 Study setting; EUCT 2023-509703-33-00

*We strongly recommend reading this statement in conjunction with the CONSORT 2010, extension to randomised pilot and feasibility trials, Explanation and Elaboration for important clarifications on all the items. If relevant, we also recommend reading CONSORT extensions for cluster randomised trials, non-inferiority and equivalence trials, non-pharmacological treatments, herbal interventions, and pragmatic trials. Additional extensions are forthcoming: for those and for up-to-date references relevant to this checklist, see www.consort-statement.org.

References

1. Granholm A, Munch MW, Meier N, et al. Empirical meropenem versus piperacillin/tazobactam for adult patients with sepsis (EMPRESS) trial: Protocol. Acta Anaesthesiol Scand 2024; 68: 1107–19.

2. Eldridge SM, Chan CL, Campbell MJ, et al. CONSORT 2010 statement: extension to randomised pilot and feasibility trials. BMJ 2016; i5239.
